# Genome sequences of six recombinant variants of potato virus Y identified in North American potato cultivars grown in China

**DOI:** 10.1128/mra.00512-23

**Published:** 2023-12-22

**Authors:** Mariana Rodriguez-Rodriguez, Mohamad Chikh-Ali, Xue Feng, Alexander V. Karasev

**Affiliations:** 1 Department of Entomology, Plant Pathology, and Nematology, University of Idaho, Moscow, Idaho, USA; 2 Shanxi Agricultural University, College of Plant Protection, Taigu, Shanxi, China; DOE Joint Genome Institute, Berkeley, California, USA

**Keywords:** potato virus Y, recombinant genome

## Abstract

Six genome sequences for potato virus Y (PVY) recombinants are reported from two North American potato cultivars grown in China. The coding complete sequences encode a single open reading frame characteristic of potyviruses. The six sequenced PVY isolates represent three distinct recombinants of PVY, namely N-Wi, SYR-I, and SYR-II.

## ANNOUNCEMENT

Potato virus Y (PVY) has flexuous filamentous particles and a positive-sense, single-stranded RNA ca. 9.7 kb genome encoding a single open reading frame (ORF) (1–4). PVY belongs to the genus *Potyvirus* (family *Potyviridae*) (2–5) and exists as a complex of strains and genetic variants including multiple, more than 35, recombinants (2, 6–9). China is one of the potato-growing countries with a great diversity of PVY recombinants described (10–12) but this diversity was addressed primarily for the local potato cultivars (10–12), and not studied in cultivars of North American origin.

In all, 26 samples from four North American potato cultivars, Blazer Russet, Ranger Russet, Russet Burbank, Shepody, and from unknown cultivar(s) were collected in June–July 2018 from potatoes originating in four provinces of China, Hebei, Inner Mongolia, Shaanxi, and Ningxia. Leaf samples from plants showing virus symptoms were collected from both seed and commercial fields and checked with the PVY-specific Immunostrip test (Agdia, Elkhart, IN). PVY-positive samples were printed on Whatman FTA PlantSaver Cards (GE Healthcare Life Sciences, Piscataway, NJ) according to the manufacturer’s instructions. The cards were dried at room temperature for 1 hour, sealed in a plastic bag with silica gel, and transported to the University of Idaho (UI) Virology Laboratory for further analysis.

**Fig 1:**
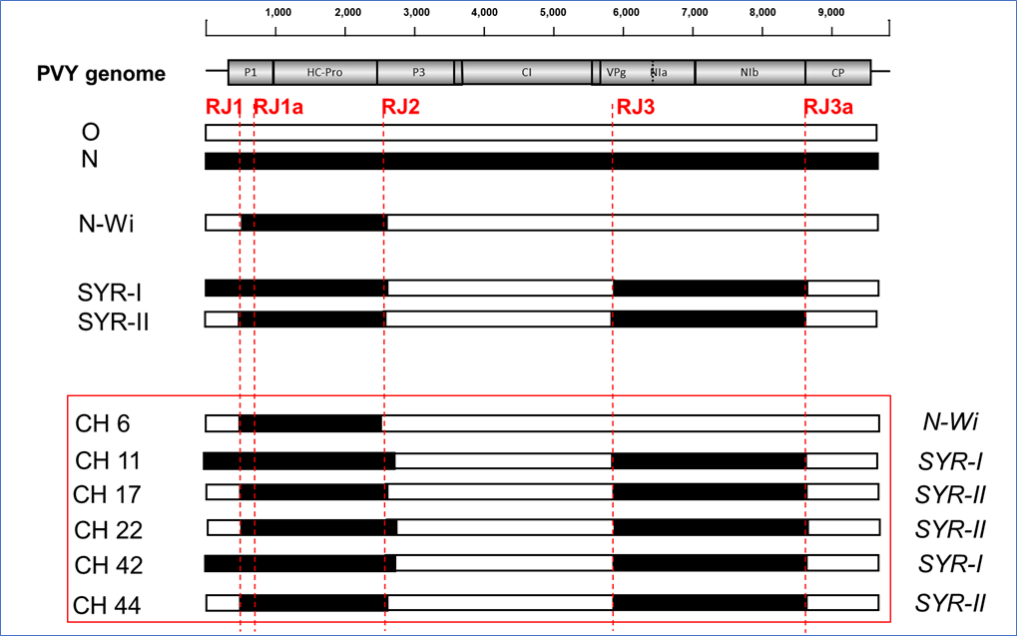
Summary diagram of the recombinant structure for the six PVY genomes identified by sequence analysis and boxed in red. Two parental sequences of PVY^O^ and PVY^Eu-N^ are presented as white and black rectangles, respectively, below the PVY genome diagram. Recombinants are presented under the parental sequences, with black and white colors indicating the origin of the segments from the two parents. Positions of the main diagnostic recombinant junctions (RJs) are marked by red lines. Three reference recombinants, N-Wi, SYR-I, and SYR-II according to reference (2), are presented above the red box for comparisons. The aligned sequences were checked for recombination using the RDP (18), GENECONV (19), BOOTSCAN (20), MAXCHI (21), CHIMAERA (22), 3Seq (23), and SiScan (24) methods in RDP4 v4.61 software (25). Recombination analysis was conducted as described (6, 7, 26), with an isolate considered to be recombinant if all six programs of the RDP package had significant support (*P* < 0.0001).

PVY strains were typed according to the methods reported in reference (13) using two multiplex RT-PCR assays (14, 15). RNA was extracted from FTA cards using RNA elution buffer and isopropanol precipitation (16). Reverse transcription (RT) PCR was done using oligo-dT and random hexamer primers (17). Control isolates representing nine strains of PVY and additional recombinant variants were from the laboratory collection (17). RT-PCR assays confirmed PVY infection in 22 out of 26 samples printed onto the FTA cards and identified all of them as recombinants by amplifications across recombination junctions (RJs) (Fig. 1).

Six of the isolates representing six banding patterns were selected for whole-genome sequencing and characterization (Table 1). The whole-genome sequencing was conducted directly on 16 overlapping RT-PCR fragments spanning the nearly complete PVY genome, using conventional methodology (6). Amplified PCR products were treated with Exosap-It (Affymetrix, Cleveland, OH) and submitted for Sanger sequencing to Elim Biopharmaceuticals (Hayward, CA). Individual sequence reads were assembled using the SeqMan Pro program of the Lasergene14 Suite (DNASTAR, Madison, WI, USA). The identity of the sequenced samples was determined using the BLASTn program provided by the National Center for Biotechnology Information. For the multiple nucleotide sequence alignment of the whole genomes, MUSCLE (27) implemented in MEGA 7 (28) was used with the default parameters.

**TABLE 1:** Genome and recombination analysis of the sequenced PVY isolates

Isolate ID	GenBank accession	Genome size, nt	GC, %	RT-PCR typing, band sizes, nt		Genome sequence typing^*c*^	Potato cultivar ^*d*^	Province
				Method 1^*a*^	Method 2^*b*^			
CH6	OR133700	9,615	42.5	181, 689	441	N-Wi	RR	Hebei
CH11	OR133701	9,609	42.0	452	441, 633	SYR-I	RR	Hebei
CH17	OR133702	9,615	42.0	181, 452	441	SYR-II	RR	Inner Mongolia
CH22	OR133703	9,618	42.0	452	441	SYR-II	Sh	Inner Mongolia
CH42	OR133704	9,615	41.9	-	441, 633	SYR-I	Sh	Shaanxi
CH44	OR133705	9,634	42.1	181	441	SYR-II	RR	Shaanxi

^*a*^ RT-PCR typing using the methodology of Lorenzen et al. (15).

^*b*^ RT-PCR typing according to Chikh-Ali et al. (17).

^*c*^ PVY strain typing according to Karasev and Gray (2) and Green et al. (7).

^*d*^ RR = Ranger Russet; Sh = Shepody.

Three recombinant types were revealed among the six sequenced PVY genomes (Fig. 1), one PVY^N-Wi^, three PVY-SYR-II, and two PVY-SYR-I, in general agreement with the PVY strain composition determined for local Chinese potato cultivars (10–12). Examination of the sequences around the two main recombinant junctions, RJ2 and RJ3, revealed the shifts for the RJ breakpoint locations in Chinese PVY recombinants which may be responsible for the unusual banding patterns observed for the RT-PCR typing (Table 1).

## Data Availability

The coding complete genome sequences of the PVY isolates determined are available in GenBank under accession numbers OR133700 (CH6), OR133701 (CH11), OR133702 (CH17), OR133703 (CH22), OR133704 (CH42), and OR133705 (CH44). The raw sequence data were deposited in the NCBI Sequence Read Archive (SRA) under BioProject accession number PRJNA994136.

## References

[1] Kerlan C. 2006. Association of applied biologists, Wellesbourne, UK. Descriptions of plant viruses. Available from: https://www.dpvweb.net/dpv/showdpv/?dpvno=414

[2] Karasev AV, Gray SM. 2013. Continuous and emerging challenges of potato virus Y in potato . Annu Rev Phytopathol 51:571–586. doi:10.1146/annurev-phyto-082712-102332 23915135

[3] Quenouille J, Vassilakos N, Moury B. 2013. Potato virus Y: a major crop pathogen that has provided major insights into the evolution of viral pathogenicity. Mol Plant Pathol 14:439–452. doi:10.1111/mpp.12024 23480826 PMC6638879

[4] Kreuze JF, Souza-Dias JAC, Jeevalatha A, Figueira AR, Valkonen JPT, Jones RAC. 2020. Viral diseases in potato. In Campos H, Ortiz O (ed), The potato crop. Springer, Cham, Switzerland.

[5] Singh RP, Valkonen JPT, Gray SM, Boonham N, Jones RAC, Kerlan C, Schubert J. 2008. Discussion paper: the naming of potato virus Y strains infecting potato. Arch Virol 153:1–13. doi:10.1007/s00705-007-1059-1 17943395

[6] Green KJ, Brown CJ, Gray SM, Karasev AV. 2017. Phylogenetic study of recombinant strains of potato virus Y. Virology 507:40–52. doi:10.1016/j.virol.2017.03.018 28399436

[7] Green KJ, Brown CJ, Karasev AV. 2018. Genetic diversity of potato virus Y (PVY): sequence analyses reveal ten novel PVY recombinant structures. Arch Virol 163:23–32. doi:10.1007/s00705-017-3568-x 28940005

[8] Green KJ, Quintero-Ferrer A, Chikh-Ali M, Jones RAC, Karasev AV. 2020. Genetic diversity of nine new non-recombinant potato virus Y isolates from three biological strain groups: historical and geographical insights. Plant Dis 104:2317–2323. doi:10.1094/PDIS-02-20-0294-SC 32692623

[9] Green KJ, Funke CN, Chojnacky J, Alvarez-Quinto RA, Ochoa JB, Quito-Avila DF, Karasev AV. 2020. Potato virus Y (PVY) isolates from Solanum betaceum represent three novel recombinants within the PVY^N^ strain group and are unable to systemically spread in potato. Phytopathology 110:1588–1596. doi:10.1094/PHYTO-04-20-0111-R 32370660

[10] Bai Y, Han S, Gao Y, Zhang W, Fan G, Qiu C, Nie X, Wen J. 2019. Genetic diversity of potato virus Y in potato production areas in northeast China. Plant Dis 103:289–297. doi:10.1094/PDIS-04-18-0687-RE 30501466

[11] Jia M-A, Wang L, Zhang J-W, Guo Y-S, Li X-D, Tian Y-P. 2021. Genetic diversity of potato virus Y potato isolates from Shandong province. J Plant Pathol 103:843–852. doi:10.1007/s42161-021-00850-0

[12] Ding P, Chen D, Feng H, Li J, Cao H, Tang M, Li J, Hao X, Han P, Meng Z, Karasev AV, Feng X. 2022. Prevalence and strain composition of potato virus Y circulating in potato fields in China’s North-central province of Shanxi. Plant Dis 106:1434–1445. doi:10.1094/PDIS-09-21-1950-RE 34813711

[13] Chikh-Ali M, Alruwaili H, Vander Pol D, Karasev AV. 2016. Molecular characterization of recombinant strains of potato virus Y from Saudi Arabia. Plant Dis 100:292–297. doi:10.1094/PDIS-05-15-0562-RE 30694145

[14] Lorenzen JH, Piche LM, Gudmestad NC, Meacham T, Shiel P. 2006. A Multiplex PCR assay to characterize potato virus Y isolates and identify strain mixtures. Plant Dis 90:935–940. doi:10.1094/PD-90-0935 30781033

[15] Chikh Ali M, Maoka T, Natsuaki T, Natsuaki KT. 2010. PVYN^TN-NW,^ a novel recombinant strain of potato virus Y predominating in potato fields in Syria. Plant Pathol 59:31–41. doi:10.1016/j.jviromet.2009.12.010

[16] Ndunguru J, Taylor NJ, Yadav J, Aly H, Legg JP, Aveling T, Thompson G, Fauquet CM. 2005. Application of FTA technology for sampling, recovery and molecular characterization of viral pathogens and virus-derived transgenes from plant tissues. Virol J 2:45. doi:10.1186/1743-422X-2-45 15904535 PMC1180858

[17] Chikh-Ali M, Gray SM, Karasev AV. 2013. An improved multiplex IC-RT-PCR assay distinguishes nine strains of potato virus Y. Plant Dis 97:1370–1374. doi:10.1094/PDIS-02-13-0161-SR 30722141

[18] Martin D, Rybicki E. 2000. RDP: detection of recombination amongst aligned sequences. Bioinform 16:562–563. doi:10.1093/bioinformatics/16.6.562 10980155

[19] Sawyer SA. 1999. GENECONV: a computer package for the statistical detection of gene conversion. Washington University, St. Louis. Online.

[20] Salminen MO, Carr JK, Burke DS, McCutchan FE. 1995. Identification of breakpoints in intergenotypic recombinants of HIV type 1 by bootscanning. AIDS Res Hum Retroviruses 11:1423–1425. doi:10.1089/aid.1995.11.1423 8573403

[21] Smith J. 1992. Analyzing the mosaic structure of genes. J Mol Evol 34:126–129. doi:10.1007/BF00182389 1556748

[22] Posada D, Crandall KA. 2001. Evaluation of methods for detecting recombination from DNA sequences: computer simulations. Proc Natl Acad Sci U S A 98:13757–13762. doi:10.1073/pnas.241370698 11717435 PMC61114

[23] Boni MF, Posada D, Feldman MW. 2007. An exact nonparametric method for Inferring mosaic structure in sequence triplets. Genetics 176:1035–1047. doi:10.1534/genetics.106.068874 17409078 PMC1894573

[24] Gibbs MJ, Armstrong JS, Gibbs AJ. 2000. Sister-scanning: a monte carlo procedure for assessing signals in recombinant sequences. Bioinform 16:573–582. doi:10.1093/bioinformatics/16.7.573 11038328

[25] Martin DP, Williamson C, Posada D. 2005. RDP2: recombination detection and analysis from sequence alignments. Bioinform 21:260–262. doi:10.1093/bioinformatics/bth490 15377507

[26] Chikh-Ali M, Rodriguez-Rodriguez M, Green KJ, Kim D-J, Chung S-M, Kuhl JC, Karasev AV. 2019. Identification and molecular characterization of recombinant potato virus Y (PVY) in potato from South Korea, PVY^NTN^ strain. Plant Dis 103:137–142. doi:10.1094/PDIS-05-18-0715-RE 30412456

[27] Edgar RC. 2004. MUSCLE: multiple sequence alignment with high accuracy and high throughput. Nucleic Acids Res 32:1792–1797. doi:10.1093/nar/gkh340 15034147 PMC390337

[28] Kumar S, Stecher G, Tamura K. 2016. MEGA7: molecular evolutionary genetics analysis version 7.0 for bigger datasets. Mol Biol Evol 33:1870–1874. doi:10.1093/molbev/msw054 27004904 PMC8210823

